# Minimum Mean Arterial Pressure and Associated Mortality Outcomes in the Cardiac Intensive Care Unit

**DOI:** 10.1016/j.jacadv.2025.102543

**Published:** 2026-01-21

**Authors:** Parth S. Patel, Garima Dahiya, Benjamin Hibbert, Dustin Hillerson, Kianoush Kashani, Jacob C. Jentzer

**Affiliations:** a Department of Internal Medicine, Mayo Clinic, Rochester, Minnesota, USA; b Department of Cardiovascular Medicine, Mayo Clinic, Rochester, Minnesota, USA; c Division of Nephrology and Hypertension and Division of Pulmonary and Critical Care Medicine, Department of Medicine, Mayo Clinic, Rochester, Minnesota, USA

**Keywords:** cardiac intensive care unit, hypotension, mean arterial pressure, minimum mean arterial pressure, mortality

## Abstract

**Background:**

Hypotension is associated with increased mortality in critical care settings; limited data exist on the *minimum* mean arterial pressure (minMAP) in cardiac critical illness.

**Objectives:**

The objective of the study was to investigate the prognostic value of the minMAP within the first 24 hours of admission to cardiac intensive care unit (CICU).

**Methods:**

This retrospective, single-center study included adult CICU patients (2007-2018). The minMAP within the first 24 hours was the exposure of interest. Patients were categorized into four minMAP groups using a classification and regression tree model. Primary outcome was in-hospital mortality; secondary, 30-day mortality. Multivariable logistic and Cox regression models were adjusted for confounders.

**Results:**

Of 11,930 patients, the median minMAP was 54 (47, 62) mm Hg, distributed as follows: ≥57 (42.4%, n = 5,053); 48 to 57 (30.5%, n = 3,635); 37 to 48 (20.1%, n = 2,392); and <37 (7.1%, n = 850). In-hospital mortality was 9.1% (n = 1,080), and 11.7% (n = 1,364) died within 30-days of CICU admission (30-day mortality 11.7% [11.1% to 12.3%] by the Kaplan-Meier method). The lower minMAP was incrementally associated with higher mortality in a continuous, reverse J-shaped manner, with the lowest mortality at 71 to 75 mm Hg. Patients with minMAP <48 mm Hg had substantially higher in-hospital (adjusted OR: 1.60 [1.36-1.88]; *P* < 0.001) and 30-day (adjusted HR: 1.47 [1.31-1.65]; *P* < 0.001) mortality. The minMAP <37 mm Hg had the highest risk in-hospital (adjusted OR: 2.19 [1.67-2.86]; *P* < 0.001) and 30-day (adjusted HR: 1.95 [1.61-2.36], *P* < 0.001) mortality. Lower minMAP was associated with higher mortality across admission diagnoses and regardless of vasoactive drugs administration.

**Conclusions:**

We observed a graded association between lower minMAP and increased mortality, re-emphasizing severe hypotension as a critical physiological marker of patient vulnerability.

Hypotension is a well-established predictor of morbidity and mortality across diverse intensive care unit (ICU) populations, including the cardiac ICU (CICU).^1^, ^2^, ^3^, ^4^ A fundamental goal of hypotension management is to maintain mean arterial pressure (MAP) above a critical threshold, often set at 65 mm Hg; below this level, decreased perfusion can result in tissue ischemia and end organ damage.^5^, ^6^, ^7^, ^8^ This is especially crucial in the CICU, where hypotensive periods can worsen already compromised cardiac function in vulnerable patients with myocardial ischemia or circulatory failure.^9^^,^^10^

Prior research on hypotension-related outcomes have employed various metrics for MAP measurement, demonstrating an increased risk of morbidity and mortality associated with severe hypotension in various intensive care settings.^1^, ^2^, ^3^, ^4^^,^^11^, ^12^, ^13^ The mean or averaged MAP measurements, although beneficial for assessing short- and long-term outcomes, may not capture brief, but critical, severe drops in blood pressure. And although transient, the sequelae from such severe hypotensive episodes may carry significant clinical consequences and adverse outcomes; however, dedicated research to include this has yet to be conducted.

Prior CICU cohort analyses, although establishing adverse outcomes with lower mean MAP, have similarly highlighted the need for dedicated research on the *minimum* MAP (minMAP).^14^, ^15^, ^16^ The effects of minMAP might differ in CICU patients, who are at risk of demand ischemia with myocardial stunning from transient hypotension and impaired forward flow with higher afterload from higher blood pressure. The mortality outcomes associated with such nadir pressures, or minMAP, remain understudied in the CICU setting. Accordingly, this study aims to address this crucial knowledge gap by investigating the mortality outcomes associated with the minMAP within the first 24 hours of CICU admission.

## Methods

### Study population

This retrospective cohort study included all consecutive, unique adult patients (aged ≥18 years) admitted to the Mayo Clinic CICU in Rochester, Minnesota, from January 2007 to April 2018. Patients were excluded if they lacked minMAP data, defined subsequently, in the first 24 hours of admission. The study was approved by the Mayo Clinic Institutional Review Board (IRB #16 to 000722) with a waiver of informed consent. Patients were excluded if they did not consent to have their data used for research purposes.

### Data sources

Patient data were extracted from the Mayo Clinic ICU DataMart, including demographic data, comorbidities, diagnoses, vital signs, laboratory values, therapies/procedures, and outcomes, as previously described.^17^ Admission laboratory values were those recorded closest to the time of CICU admission. Admission diagnoses included all International Classification of Diseases-9/10 codes documented within 1 day of CICU admission and were not mutually exclusive.^18^ The Charlson Comorbidity Index, Acute Physiology and Chronic Health Evaluation III, and Sequential Organ Failure Assessment scores were calculated using validated electronic algorithms.^19^^,^^20^ Mayo CICU Admission Risk Score was calculated using data from the time of admission; notably, this validated CICU risk score outperforms Acute Physiology and Chronic Health Evaluation III and Sequential Organ Failure Assessment in this population and does not include vital sign data.^21^

### Exposures of interest and outcomes

The primary exposure was minMAP, defined as the lowest MAP recorded within the first 24 hours of CICU admission. MAP was measured either invasively via an arterial catheter or noninvasively via a blood pressure cuff. Continuous vital signs were recorded at 5 to 15 minute intervals and uploaded automatically to the ICU DataMart after validation by the bedside nurse to exclude obviously erroneous values or incorrect measurements. The minMAP data were extracted from these measurements.

Patients were categorized into groups based on minMAP using a classification and regression tree (CART) model for prediction of in-hospital mortality. We sought to identify multiple potential minMAP cut-offs that could identify groups with higher and lower risk of in-hospital mortality in a data-driven manner within our population. Accordingly, patients were categorized into 4 groups based on minMAP using a CART model for prediction of in-hospital mortality with 3 nodes. CART is a nonparametric machine learning technique that constructs a binary decision tree. The algorithm recursively partitions data by evaluating all possible splits to identify the optimal variable and cutoff at each node. This process aims to maximize the separation of the outcome variable, which in this study was in-hospital mortality, between the resulting subgroups. Discrimination of logistic regression models was evaluated using the area under the receiver operator characteristic curve (AUC) C-statistics. The primary outcome of interest was all-cause in-hospital mortality, inclusive of CICU mortality, and the secondary outcome was all-cause 30-day mortality. Further subgroup analyses were conducted according to admission diagnoses and critical care diagnoses, defined as diagnoses qualifying for intensive care unit hospitalization, and therapies. The presence of acute kidney injury (AKI) was determined based on admission and maximum creatinine values during hospitalization and the need for new dialysis based on the modified Kidney Disease Improving Global Outcomes criteria.

### Statistical analysis

Categorical variables were summarized as n (%) and groups were compared using chi-square tests. Continuous variables were summarized as median (IQR), and groups were compared using Wilcoxon/Kruskal-Wallis rank-sum tests. Predictors of in-hospital mortality were evaluated using logistic regression, with OR and 95% CI values calculated before and after multivariable adjustment. Kaplan-Meier curves were used to evaluate 30-day survival, with groups compared using the log-rank test. Predictors of 30-day mortality were evaluated using Cox proportional-hazards models, with HR and 95% CI values calculated before and after multivariable adjustment. The proportional-hazards assumption for Cox models was tested using Schoenfeld residual plots; however, violations of the proportional-hazards assumptions were noted. Multivariable models were adjusted for the following variables selected a priori based on clinical relevance and known associations with mortality in this population: age, sex, race, Charlson Comorbidity Index , Mayo CICU Admission Risk Score , number of vasoactive drugs, lowest Glasgow Coma Scale during the first 24 hours, diagnosis of acute coronary syndrome or heart failure, in-hospital cardiac arrest, and the use of cardiac catheterization/percutaneous coronary intervention, positive-pressure ventilation, pulmonary artery catheters, mechanical circulatory support, and dialysis. Sensitivity analyses were performed after excluding patients with a CICU length of stay <1 day and after excluding early CICU deaths, defined as deaths within 1 day. All statistical analyses were performed using BlueSky (BlueSky Statistics, version 10.3.1 Pro).

## Results

### Study population

Of 12,428 unique patients in our CICU database, 11,930 had available minMAP data and were included in this study. The median age was 69.4 (58.1, 79.2) years, and 37.7% (n = 4,500) were females. Admission diagnoses included heart failure (48.7%, n = 5,757), acute coronary syndrome (42.7%, n = 5,048), shock (14.9%, n = 1767), and cardiac arrest (11.8%, n = 1,397). Critical care therapies, inclusive of hemodynamic/ventilatory support or continuous renal replacement therapy, were used in 58.5% (n = 6,982), including vasoactive drugs in 24.9% (n = 2,969) and positive-pressure ventilation in 28.1% (n = 3,356). The median minMAP was 54 (47, 62) mm Hg. The remainder of the demographic data are shown in Table 1.

**Table 1:** Baseline Demographics Stratified by minMAP Group

Minimum MAP CART Group	≥57 (N = 5,053)	48-57 (N = 3,635)	37-48 (N = 2,392)	<37 (N = 850)	Total (N = 11,930)	*P* Value
Age, y	66.5 (55.1, 77.3)	71.1 (60.3, 80.4)	72.7 (61.6, 81.5)	68.5 (59.1, 77.0)	69.4 (58.1, 79.2)	<0.001
Female	1,604 (31.7%)	1,502 (41.3%)	1,053 (44.0%)	341 (40.1%)	4,500 (37.7%)	<0.001
Caucasian ethnicity	4,629 (91.6%)	3,390 (93.3%)	2,231 (93.3%)	765 (90.0%)	11,015 (92.3%)	<0.001
ICU length of stay	1.5 (0.9, 2.5)	1.8 (1.0, 3.0)	1.9 (1.0, 3.3)	1.8 (0.8, 3.7)	1.7 (0.9, 2.9)	<0.001
Hospital length of stay	3.8 (2.4, 7.2)	5.0 (2.9, 9.4)	5.7 (3.0, 10.2)	6.0 (2.7, 12.0)	4.6 (2.7, 8.9)	<0.001
APACHE III scores	48.0 (36.0, 61.0)	62.0 (50.0, 75.0)	68.0 (54.0, 84.0)	71.0 (57.0, 93.0)	58.0 (44.0, 73.0)	<0.001
Charlson Comorbidity Index scores	1.0 (0.0, 3.0)	2.0 (0.0, 4.0)	2.0 (0.0, 4.0)	2.0 (0.0, 4.0)	2.0 (0.0, 4.0)	<0.001
Day 1 SOFA scores	2.0 (1.0, 3.0)	3.0 (1.0, 5.0)	4.0 (2.0, 7.0)	5.0 (2.0, 8.0)	2.0 (1.0, 5.0)	<0.001
MCARS scores	1.0 (0.0, 2.0)	2.0 (1.0, 3.0)	2.0 (1.0, 4.0)	3.0 (1.0, 5.0)	2.0 (0.0, 3.0)	<0.001
Prior history of MI	828 (16.4%)	746 (20.6%)	479 (20.0%)	152 (17.9%)	2,205 (18.5%)	<0.001
Prior history of CHF	788 (15.6%)	830 (22.9%)	587 (24.6%)	210 (24.7%)	2,415 (20.3%)	<0.001
Prior history of stroke	540 (10.7%)	433 (11.9%)	350 (14.6%)	105 (12.4%)	1,428 (12.0%)	<0.001
Prior history of diabetes mellitus	1,278 (25.4%)	1,077 (29.7%)	783 (32.8%)	266 (31.3%)	3,404 (28.6%)	<0.001
Prior history of liver disease	83 (1.6%)	71 (2.0%)	69 (2.9%)	23 (2.7%)	246 (2.1%)	0.003
Prior history of cancer	975 (19.4%)	843 (23.2%)	546 (22.8%)	161 (19.0%)	2,525 (21.2%)	<0.001
Prior history of lung disease	864 (17.2%)	766 (21.1%)	511 (21.4%)	168 (19.8%)	2,309 (19.4%)	<0.001
Admission diagnosis						
Sepsis	152 (3.0%)	247 (6.8%)	248 (10.5%)	103 (12.2%)	750 (6.3%)	<0.001
Respiratory failure	818 (16.4%)	977 (27.0%)	752 (31.7%)	326 (38.5%)	2,873 (24.3%)	<0.001
Acute coronary syndrome	2,167 (43.3%)	1,482 (41.0%)	1,034 (43.6%)	365 (43.1%)	5,048 (42.7%)	0.123
Heart failure	2025 (40.5%)	1860 (51.5%)	1,361 (57.4%)	511 (60.3%)	5,757 (48.7%)	<0.001
CKD	862 (17.1%)	790 (21.8%)	585 (24.5%)	212 (25.0%)	2,449 (20.6%)	<0.001
Cardiac arrest	446 (8.9%)	418 (11.6%)	354 (14.9%)	179 (21.1%)	1,397 (11.8%)	<0.001
Shock	374 (7.5%)	516 (14.3%)	554 (23.4%)	323 (38.1%)	1767 (14.9%)	<0.001
In-hospital LVEF	52.0 (39.0, 61.0)	50.0 (35.0, 60.0)	50.0 (33.0, 60.0)	45.0 (27.0, 60.0)	50.0 (35.0, 60.0)	<0.001
Cardiogenic shock stage						<0.001
A	2,512 (60.2%)	1,183 (40.3%)	531 (28.8%)	114 (20.6%)	4,340 (45.6%)	
B	942 (22.6%)	1,035 (35.2%)	706 (38.2%)	205 (37.1%)	2,888 (30.4%)	
C	626 (15.0%)	500 (17.0%)	322 (17.4%)	58 (10.5%)	1,506 (15.8%)	
D	87 (2.1%)	205 (7.0%)	255 (13.8%)	135 (24.4%)	682 (7.2%)	
E	5 (0.1%)	16 (0.5%)	32 (1.7%)	41 (7.4%)	94 (1.0%)	
KDIGO AKI						<0.001
No AKI	3,626 (75.8%)	2,328 (67.8%)	1,368 (62.4%)	445 (58.9%)	7,767 (69.6%)	
Stage 1	885 (18.5%)	816 (23.8%)	563 (25.7%)	180 (23.8%)	2,444 (21.9%)	
Stage 2	87 (1.8%)	103 (3.0%)	87 (4.0%)	31 (4.1%)	308 (2.8%)	
Stage 3	184 (3.8%)	188 (5.5%)	173 (7.9%)	99 (13.1%)	644 (5.8%)	
In-hospital dialysis	134 (2.7%)	154 (4.2%)	162 (6.8%)	104 (12.2%)	554 (4.6%)	<0.001
Any critical care therapy use	2,444 (48.4%)	2,231 (61.4%)	1,641 (68.6%)	666 (78.4%)	6,982 (58.5%)	<0.001
Any ventilator use	1,004 (19.9%)	1,133 (31.2%)	866 (36.2%)	353 (41.5%)	3,356 (28.1%)	<0.001
Invasive mechanical ventilation	442 (8.7%)	662 (18.2%)	580 (24.2%)	270 (31.8%)	1954 (16.4%)	<0.001
Vasoactive use	624 (12.3%)	938 (25.8%)	950 (39.7%)	457 (53.8%)	2,969 (24.9%)	<0.001
CRRT	21 (0.4%)	61 (1.7%)	99 (4.1%)	56 (6.6%)	237 (2.0%)	<0.001
Dialysis	134 (2.7%)	154 (4.2%)	162 (6.8%)	104 (12.2%)	554 (4.6%)	<0.001
Underwent cardiac catheterization	3,053 (60.4%)	2018 (55.5%)	1,359 (56.8%)	503 (59.2%)	6,933 (58.1%)	<0.001
Underwent PCI	1846 (36.5%)	1,238 (34.1%)	810 (33.9%)	287 (33.8%)	4,181 (35.0%)	0.036
First 24 hours min MAP in the ICU	64.0 (60.0, 69.0)	52.0 (50.0, 54.0)	43.0 (40.0, 46.0)	33.0 (32.0, 35.0)	54.0 (47.0, 62.0)	<0.001
First 24 hours max MAP in the ICU	106.0 (96.0, 119.0)	101.0 (90.0, 117.0)	104.0 (90.0, 122.0)	116.0 (95.0, 149.0)	105.0 (93.0, 120.0)	<0.001
First 24 hours min HR in the ICU	55.0 (43.0, 64.0)	52.0 (40.0, 63.0)	49.0 (36.0, 61.0)	45.0 (34.0, 59.0)	53.0 (40.0, 63.0)	<0.001
First 24 hours max HR in the ICU	101.0 (89.0, 118.0)	106.0 (91.0, 125.0)	110.0 (93.0, 133.0)	120.0 (101.0, 143.0)	105.0 (91.0, 125.0)	<0.001
First 24 hours min RR in the ICU	10.0 (7.0, 12.0)	9.0 (6.0, 12.0)	9.0 (6.0, 12.0)	8.0 (6.0, 11.0)	9.0 (7.0, 12.0)	<0.001
First 24 hours max RR in the ICU	28.0 (24.0, 33.0)	30.0 (26.0, 36.0)	31.0 (27.0, 38.0)	32.0 (27.0, 40.0)	29.0 (25.0, 35.0)	<0.001
First 24 hours min temperature in the ICU	36.5 (36.3, 36.6)	36.4 (36.3, 36.6)	36.4 (36.0, 36.6)	36.3 (35.5, 36.6)	36.4 (36.3, 36.6)	<0.001
First 24 hours max temperature in the ICU	37.0 (36.8, 37.3)	37.0 (36.8, 37.5)	37.1 (36.8, 37.6)	37.0 (36.8, 37.6)	37.0 (36.8, 37.4)	<0.001

APACHE III = acute physiology and chronic health evaluation III; CART = classification and regression tree; CHF = congestive heart failure; CKD = chronic kidney disease; CRRT = continuous renal replacement therapy; HR = heart rate; ICU = intensive care unit; KDIGO = kidney disease improving global outcomes; LVEF = left ventricular ejection fraction; MAP = mean arterial pressure; MCARS = Mayo-CICU admission risk score; MI = myocardial infarction; minMAP = minimum MAP; PCI = percutaneous coronary intervention; RR = respiratory rate rate; SOFA = sequential organ failure assessment.

### Minimum MAP groups

CART analysis identified primary and secondary nodal points. The primary node, identifying the optimal mortality cutoff, was at 48 mm Hg, with subsequent secondary nodes at 37 mm Hg and 57 mm Hg. This yielded 4 groups: minMAP ≥57 mm Hg, 48 to 57 mm Hg, 37 to 48 mm Hg, and <37 mm Hg (Central Illustration). The distribution of minMAP groups was as follows: ≥57 (42.4%, n = 5,053); 48 to 57 (30.5%, n = 3,635); 37 to 48 (20.1%, n = 2,392); and <37 (7.1%, n = 850). Baseline characteristics differed substantially according to minMAP group, including a higher severity of illness, more critical care diagnoses, and higher use of critical care therapies in the lower minMAP groups (Table 1). Prevalence and severity of AKI increased with lower minMAP (Table 1).

**Central Illustration:**
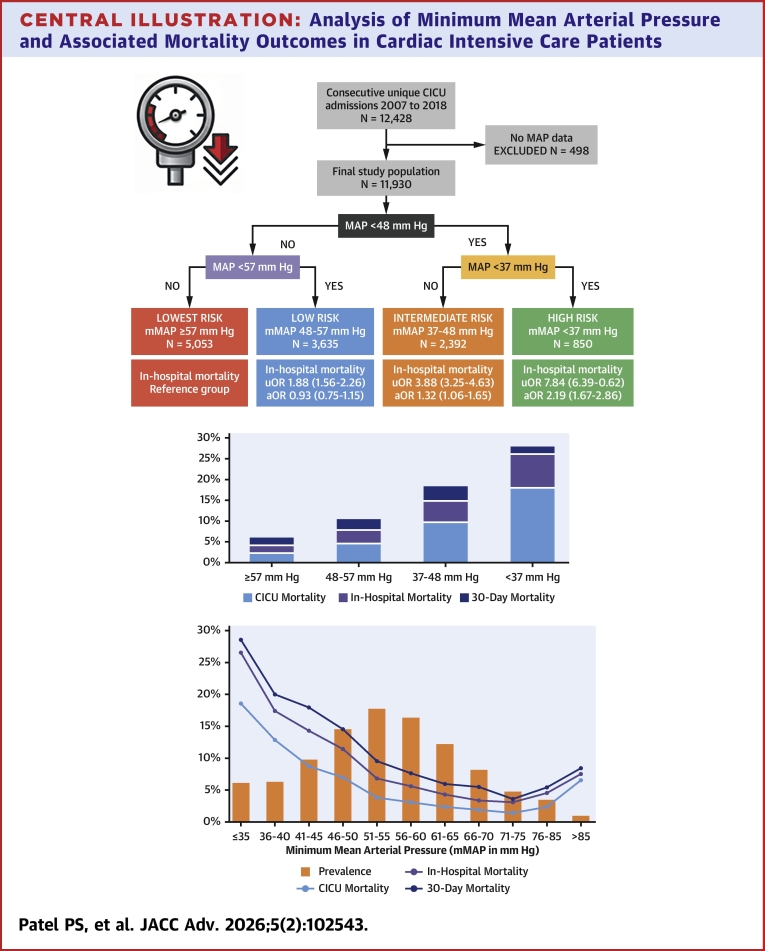
Analysis of Minimum Mean Arterial Pressure and Associated Mortality Outcomes in Cardiac Intensive Care Patients Flow diagram demonstrating study population and minMAP groups, with associated unadjusted OR (uOR) and adjusted OR (aOR) in-hospital mortality and histogram showing minMAP distributions, optimal cutoff by CART, and associated CICU, in-hospital, and 30-day mortality. CICU = cardiac intensive care unit; minMAP = minimum mean arterial pressure.

### In-hospital mortality

A total of 1,080 (9.1%) patients died during hospitalization, including 672 (5.6%) who died in the CICU (Table 2). The median minMAP was lower in patients who died in the CICU (46 vs 55 mm Hg, *P* < 0.001) and during the hospitalization (47 vs 55 mm Hg, *P* < 0.001). Both CICU and in-hospital mortality increased incrementally with lower minMAP in a continuous, reverse J-shaped manner (Central Illustration). The lowest mortality was observed at a minMAP of 71 to 75 mm Hg in the overall cohort. Each 5 mm Hg increase in minMAP was associated with lower in-hospital mortality (unadjusted OR: 0.74 [0.72-0.76]; *P* < 0.001), and even after adjustment (adjusted OR: 0.91 [0.88-0.95]; *P* < 0.001), until the nadir mortality at minMAP 71 to 75 mm Hg. Both CICU and in-hospital mortality increased in a stepwise manner in each lower minMAP group (Central Illustration). Patients with minMAP <48 mm Hg had a substantially higher risk before (unadjusted OR: 3.54 [3.12-4.02]; *P* < 0.001) and after (adjusted OR: 1.60 [1.36-1.88]; *P* < 0.001) adjustment. Patients with minMAP <37 mm Hg had the highest risk of in-hospital mortality, followed by those with minMAP 37 to 48 mm Hg; patients with minMAP 48 to 57 mm Hg did not have a higher risk after adjustment (Central Illustration).

**Table 2:** In-Hospital, CICU, and 30-Day Mortality Stratified by minMAP Group

Minimum MAP CART Group	≥57 (n = 5,053)	48-57 (n = 3,635)	37-48 (n = 2,392)	<37 (n = 850)	Total (N = 11,930)	*P* Value
CICU death	120 (2.4%)	167 (4.6%)	232 (9.7%)	153 (18.0%)	672 (5.6%)	<0.001
Hospital death	218 (4.3%)	284 (7.8%)	356 (14.9%)	222 (26.1%)	1,080 (9.1%)	<0.001
30-day mortality KM estimates	6.1% (5.5-6.9%)	10.8% (9.7%-11.8%)	18.8% (17.2%-20.3%)	28.3% (25.2%-31.2%)	11.7% (11.1-12.3%)	<0.001

CICU = cardiac intensive care unit; KM = Kaplan-Meier; other abbreviations as in Table 1.

**Figure 1:**
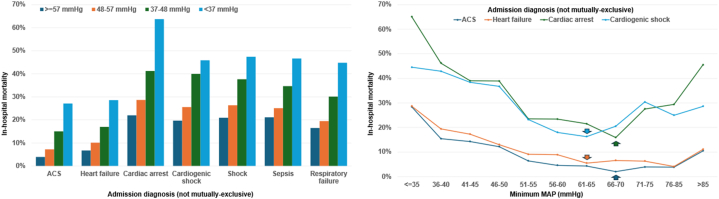
In-Hospital Mortality According to minMAP Group and Admission Diagnosis With Nadir Mortality ACS = acute coronary syndrome; MAP = mean arterial pressure.

**Figure 2:**
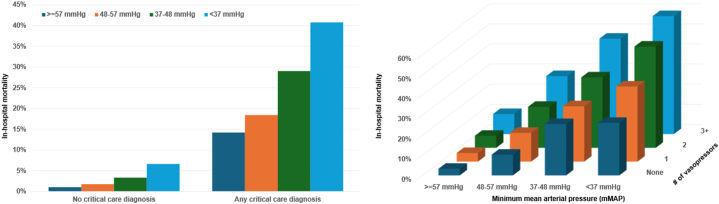
In-Hospital Mortality According to minMAP Group and Critical Care Diagnosis or Number of Vasopressors

Patients in lower minMAP groups had higher mortality across admission diagnosis subgroups (Figure 1). The minMAP value associated with the nadir mortality was slightly higher for acute coronary syndrome or cardiac arrest patients (66-70 mm Hg) than for heart failure or cardiogenic shock patients (61-65 mm Hg) (Figure 1). Each 5 mm Hg increase in minMAP was associated with lower in-hospital mortality in patients with (unadjusted OR: 0.81 [0.79-0.84]; AUC: 0.64 [0.62-0.66]) and without (unadjusted OR: 0.76 [0.71-0.82]; AUC: 0.67 [0.62-0.71]) critical care diagnoses. The strength of association between minMAP and in-hospital mortality (unadjusted OR per 5 mm Hg higher) varied by admission diagnosis: acute coronary syndrome, 0.72 (0.69-0.76); heart failure, 0.77 (0.74-0.80); cardiogenic shock, 0.82 (0.78-0.87); sepsis, 0.82 (0.75-0.89); and cardiac arrest, 0.80 (0.76-0.84). Patients who received vasopressors had higher mortality in each minMAP group, with a gradient of risk according to MAP and number of vasopressors (Figure 2). Each 5 mm Hg increase in minMAP was associated with lower in-hospital mortality in patients who did (unadjusted OR: 0.80 [0.76-0.84]; AUC: 0.63 [0.61-0.66]) and did not (unadjusted OR: 0.87 [0.83-0.91]; AUC: 0.59 [0.56-0.62]) receive vasopressors. The strength of association between minMAP and in-hospital mortality varied across minMAP groups: minMAP ≥57 mm Hg (unadjusted OR: 0.99 per 5 mm Hg higher; 95% CI: 0.90-1.08), 48 to 57 mm Hg (unadjusted OR: 0.70 per 5 mm Hg higher; 95% CI: 0.55-0.89), 37 to 48 mm Hg (unadjusted OR: 0.85 per 5 mm Hg higher; 95% CI: 0.71-1.02), and <37 mm Hg (unadjusted OR: 0.64 per 5 mm Hg higher; 95% CI: 0.41-1.00).

### Thirty-day mortality

**Figure 3:**
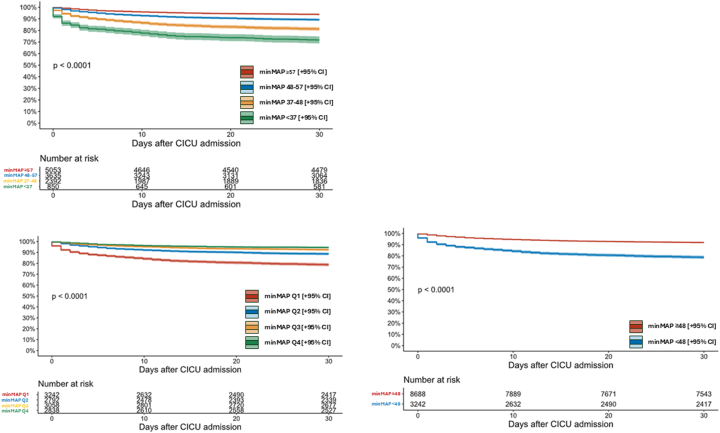
Kaplan-Meier Curve Demonstrating 30-Day Mortality by minMAP CART Group, minMAP Quartile, and minMAP 48 mm Hg Cutoff CICU = cardiac intensive care unit; minMAP = minimum mean arterial pressure.

A total of 1,364 (11.7%) patients died within 30 days of CICU admission, including in-hospital deaths and deaths after discharge (Table 2, Supplemental Figure 1). Thirty-day mortality was higher in the lower MAP groups (Figure 3, Supplemental Figure 1); each 5 mm Hg higher minMAP was associated with decreased 30-day mortality (unadjusted HR: 0.77 [0.76-0.79]; *P* < 0.001), even after adjustment (adjusted HR: 0.91 [0.89-0.94]; *P* < 0.001). Patients with minMAP <48 mm Hg had a substantially higher risk before (unadjusted HR: 2.88 [2.59-3.21]; *P* < 0.001) and after (adjusted HR: 1.47 [1.31-1.65]; *P* < 0.001) adjustment. Patients with minMAP <37 mm Hg had the highest risk, followed by those with minMAP 37 to 48 mm Hg; patients with minMAP 48 to 57 mm Hg did not have a higher risk after adjustment (Figure 3).

### Sensitivity analysis

A total of 3,324 (27.9%) patients had an ICU length of stay <1 day, including 275 (8.3%) early CICU deaths which accounted for 40.9% of all CICU deaths (Supplemental Table 1). After excluding patients with a length of stay <1 day or early CICU deaths, a similar stepwise increase in CICU, in-hospital, and 30-day mortality was seen in each lower MAP group (Supplemental Table 1, Supplemental Figure 1); mortality was higher for those with a length of stay <1 day due to inclusion of early CICU deaths.

## Discussion

This study investigated the relationship between the minMAP within the first 24 hours of admission and subsequent mortality in a large, heterogenous cohort of 11,930 CICU patients. Our principal findings demonstrate a significant and graded association between lower minMAP and higher in-hospital and 30-day mortality. This association was consistently observed across various subgroups, including after stratification for admission diagnosis and use of vasoactive drugs.

### Primary findings

Through CART modeling, our analysis identified key minMAP primary and secondary nodal points of 48 mm Hg and 37 and 57 mm Hg, respectively. Patients with a minMAP <48 mm Hg had substantially higher adjusted in-hospital and 30-day mortality, with the highest mortality in the lowest minMAP group of <37 mm Hg. This 48 mm Hg threshold had the most pronounced change in mortality risk and emerged as an independent predictor of adverse outcomes. Although it is likely that the specific numerical values we identified would vary in different populations, our findings highlight the incremental hazards associated with more severe hypotension and the likelihood of nonlinear effects. Accordingly, we do not advocate for use of these specific minMAP values to guide clinical care per se and instead to underscore the need to avoid hypotension. This is particularly salient when considering the possibility that minor measurement errors for patients with MAP near a cutoff could result in reclassification into a different risk group, despite small differences not being truly meaningful for a continuous physiological parameter such as MAP.

This observation is consistent with prior studies, which have established that hypotension, defined by averaged or mean MAP values, is a significant predictor of morbidity and mortality in various intensive care settings.^1^, ^2^, ^3^, ^4^^,^^11^, ^12^, ^13^ Similarly, in the CICU, a lower MAP has been associated with higher mortality.^14^, ^15^, ^16^ Our findings reinforce these prior studies and provide new insights by demonstrating that minMAP, which may additionally reflect transient episodes of severe hypotension, is independently associated with an increased risk of mortality. Furthermore, increased mortality with lower minMAP was consistently observed across admission diagnoses. Interestingly, for patients with circulatory failure (ie, heart failure or cardiogenic shock), the minMAP range with the lowest in-hospital mortality was slightly lower than for those with acute coronary syndromes or cardiac arrest. This finding may reflect an optimal hemodynamic state between maintaining adequate organ perfusion and avoiding excessive afterload in the setting of compromised cardiac function.^22^

Our findings highlight that severe hypotension is a medical emergency conferring substantial risk across the spectrum of cardiac critical illness. The detrimental effect of lower minMAP was evident in both patients who did and did not receive vasopressors, although mortality rates were predictably higher in those requiring vasopressor support. This suggests that the nadir MAP could be an important risk factor for mortality, even when accounting for known predictors. These observations emphasize the need for diligent medication titration to prevent severe hypotensive episodes. The strong prognostic signal of minMAP, even in vasopressor-dependent shock, suggests that dynamic monitoring of arterial pressure waveforms, perhaps through closed-loop vasopressor titration systems leveraging machine learning to predict drops in MAP, could play a vital role in real-time hemodynamic management and avoidance of severe hypotension.^23^, ^24^, ^25^

Interestingly, we also observed a continuous, reverse J-shaped relationship between minMAP and mortality (Central Illustration). Our data showed that mortality rates decreased with each 5 mm Hg incremental increase in minMAP up until 71 to 75 mm Hg, after which mortality began to increase. The observation that mortality begins to rise significantly at pressures below 70 to 75 mm Hg aligns with the physiological concept of autoregulatory failure during hypotension, where blood flow to vital organs becomes pressure-dependent.^26^ The observation of a reverse J-shaped relationship mirrors a prior study which similarly noted a bidirectional relationship between MAP and outcomes in patients with acute myocardial infarction.^27^ The potential risks of excessively high MAP warrant further investigation, particularly given that patients with critical cardiac illnesses are often sensitive to afterload, with high MAP predisposing them to congestion and reduced forward flow.

Current guidelines recommend MAP targets near 65 mm Hg for various critical illnesses.^5^, ^6^, ^7^, ^8^ Our analysis found that the association between minMAP and mortality differed across common CICU admission diagnoses, suggesting a need to define individualized MAP targets based on the underlying condition and patient-specific factors. This observation raises the question of whether a lower MAP target could be more advantageous for patients with circulatory failure.^22^ However, further research is needed to confirm these preliminary findings, as recorded minMAP is not synonymous with a targeted MAP goal. Ongoing trials, such as NOR-SHOCK (Clinical Outcome and Cost-effectiveness of Reduced Noradrenaline by Using a Lower Blood Pressure Target in Patients With Cardiogenic Shock From Acute Myocardial Infarction), are currently investigating this further.^28^ Although previous randomized trials have not demonstrated a survival benefit with MAP targets >65 to 70 mm Hg, some studies have reported improved renal outcomes in patients with septic shock who received a higher MAP target at the cost of higher vasopressor doses with excess cardiovascular toxicity.^29^ This divergence in outcomes is further supported by our study's findings, which show a higher incidence and severity of AKI with a lower minMAP. This observation highlights the kidneys' sensitivity to hypotension and suggests that cardiovascular, renal, and mortality outcomes may respond differently as a function of MAP.^30^^,^^31^

### Implications for predictive analysis

Machine learning predictive analysis has recently emerged as a promising avenue for the early recognition and prevention of impending hypotension in critically ill patients.^32^ These algorithms analyze high-frequency physiological data, such as arterial pressure waveforms and vital signs, to identify subtle precursors to hypotensive episodes, often before overt clinical signs appear. Such models have demonstrated high sensitivity and specificity in predicting hypotension minutes to an hour in advance, enabling earlier intervention and potentially improving patient outcomes.^33^, ^34^, ^35^ The American Heart Association's 2024 Scientific Statement further underscores this potential, noting that machine learning-based hypotension prediction can outperform traditional monitoring, particularly in less intensively monitored settings, and may reduce adverse outcomes when combined with timely intervention.^32^ Our study's identification of minMAP nodal points associated with progressively increasing mortality risk may provide potential starting data for training advanced predictive analytics in critical care. Algorithms designed to process continuous physiological data can leverage these thresholds to identify patients at high risk of impending severe hypotensive episodes who might benefit from preventative interventions. The emphasis on early recognition and prediction of hypotension is paramount, as our findings redemonstrate that the occurrence of severe hypotension, as measured by minMAP, is associated with substantially increased mortality. Timely identification, facilitated by such predictive models, could enable clinicians to implement rapid interventions before a patient's condition deteriorates to critically low MAP levels. This proactive approach holds the potential to mitigate prolonged severe hypotension and ultimately improve patient outcomes.

### Study Limitations

Several limitations of our study should be considered. As a retrospective, single-center analysis, our findings may not be generalizable to other populations and are susceptible to unmeasured confounding. In this study, minMAP was defined based on recorded values. Despite validation of MAP values by the bedside nurses, we could not confirm the accuracy of minMAP or exclude the possibility that erroneous values could have been entered automatically into the database. We were unable to differentiate between patients who had invasive vs noninvasive MAP measurements. Noninvasive measurements, particularly at MAP <60 mm Hg, have been shown to carry less accuracy and greater variability in MAP measurement, and this may have introduced potential uncertainty into our results.^36^^,^^37^ We could not determine the duration of the hypotension as the minMAP only records the lowest recorded value; we hypothesize that the “area under the curve” of hypotension, reflecting both the magnitude and duration, is the most important prognostic marker in this context. Furthermore, less frequent readings in this group may not have captured every possible hypotensive episode between measurements as accurately as invasive measurements, which are imperfect and susceptible to waveform dampening and improper leveling. Central venous pressure data were not consistently collected in a standardized manner in our cohort, thereby limiting its inclusion and precluding us from calculating mean perfusion pressure, which is a potentially more informative hemodynamic marker.^38^ In addition, the MAP target was determined by the treatment team and could have varied according to the patient, provider, and time. We adjusted our multivariable models for an extensive array of clinical variables including treatments provided during the CICU and hospital stay which could have occurred during or after the minMAP exposure period, potentially introducing an important source of bias in our effort to be comprehensive; without time stamps, we could not perform time-varying exposure analysis. Several of these covariates were likely to be directly affected by minMAP (eg, vasopressor usage) and could have been intermediary variables or effect mediators affecting associations between minMAP and mortality. Due to violations of the proportional-hazards assumptions, our Cox model results (especially the HR point estimates) should be interpreted with caution. Our study does not provide direct evidence for specific interventions aimed at targeting a specific minMAP goal per se, but rather aims at identifying the clinical outcomes associated with different minMAP values. We did not have data regarding vasopressor dosing at the time that the minMAP value occurred.

Despite the limitations, our study provides compelling evidence for the prognostic significance of minMAP within the first 24 hours of CICU admission. The strong, graded association between minMAP and mortality highlights the importance of vigilant blood pressure monitoring and the prompt management of even transient hypotensive episodes in this high-risk patient population. Our focused analysis on the nadir pressure itself, rather than mean pressure or blood pressure variability, provides a new perspective and a valuable, easily extractable metric for risk stratification. Future research should focus on validating these findings and investigating whether interventions aimed at preventing or rapidly correcting a low minMAP can improve outcomes in CICU patients, potentially using enhanced monitoring or implementation of a predictive algorithm.

## Conclusions

Our study, investigating minMAP and mortality outcomes among CICU patients, identified a strong and graded association between lower minMAP and increased mortality, a relationship that persisted across various subgroups. The minMAP data offer a critical window into the most severe periods of hemodynamic instability, highlighting the importance of vigilant blood pressure monitoring and the swift management of even transient hypotensive episodes. The identification of key minMAP thresholds associated with escalating mortality risk not only provides a valuable metric for risk stratification but also offers the potential to aid in developing advanced predictive analytics and guiding future trials focused on preventing and correcting severe hypotension in this vulnerable patient population.

## Funding support and author disclosures

The authors have reported that they have no relationships relevant to the contents of this paper to disclose.
