# Supplementary material for: Minimum Mean Arterial Pressure and Associated Mortality Outcomes in the Cardiac Intensive Care Unit
Source: JACC Adv. 2026 Jan 21;5(2):102543. doi: 10.1016/j.jacadv.2025.102543 (PMC12859509; doi:10.1016/j.jacadv.2025.102543)
Supplement: Supplementary Tables 1 and 2 and Supplementary Figures 1 and 2 [file mmc1.pdf]

Supplemental Material

Minimum Mean Arterial Pressure and Associated Mortality Outcomes in the Cardiac Intensive Care Unit

Supplemental Table 1: Sensitivity Analysis Accounting for Mortality Within The First 24 Hours of ICU Length of Stay.

ICU Length of Stay	Minimum MAP CART Group	>=57 (N=5053)	48-57 (N=3635)	37-48 (N=2392)	<37 (N=850)	Total (N=11930)	p-value
>1 Day	CICU Death	88 (2.6%)	123 (4.4%)	127 (7.0%)	59 (10.1%)	397 (4.6%)	< 0.001
	Hospital Death	159 (4.6%)	212 (7.6%)	223 (12.3%)	100 (17.1%)	694 (8.1%)	< 0.001
	Mortality at 30 Days	228 (6.6%)	291 (10.5%)	294 (16.3%)	117 (20.0%)	930 (10.8%)	< 0.001
<1 Day	CICU Death	32 (2.0%)	44 (5.1%)	105 (18.0%)	94 (35.6%)	275 (8.3%)	< 0.001
	Hospital Death	59 (3.6%)	72 (8.4%)	133 (22.8%)	122 (46.2%)	386 (11.6%)	< 0.001
	Mortality at 30 Days	76 (4.7%)	91 (10.6%)	146 (25.0%)	121 (45.8%)	434 (13.1%)	< 0.001

Timing of Death	Minimum MAP CART Group	>=57 (N=5053)	48-57 (N=3635)	37-48 (N=2392)	<37 (N=850)	Total (N=11930)	p value
Not Early Death	CICU Death	88 (1.8%)	123 (3.4%)	127 (5.6%)	59 (7.8%)	397 (3.4%)	< 0.001
	Hospital Death	186 (3.7%)	240 (6.7%)	251 (11.0%)	128 (16.9%)	805 (6.9%)	< 0.001
	Mortality at 30 Days	272 (5.4%)	338 (9.4%)	335 (14.6%)	144 (19.0%)	1089 (9.3%)	< 0.001

Early Death*	CICU Death	32 (100.0%)	44 (100.0%)	105 (100.0%)	94 (100.0%)	275 (100.0%)	
	Hospital Death	32 (100.0%)	44 (100.0%)	105 (100.0%)	94 (100.0%)	275 (100.0%)	
	Mortality at 30 Days	32 (100.0%)	44 (100.0%)	105 (100.0%)	94 (100.0%)	275 (100.0%)	

*Early deaths defined as deaths within 24 hours of admission.

Supplemental Table 2: In-Hospital Mortality According Admission Diagnosis Stratified minMAP Group

Minimum MAP CART Group	>=57 (N=5000)	48-57 (N=3612)	37-48 (N=2372)	<37 (N=847)	Total (N=11831)	p-value
Cardiac Arrest						
No	120 (2.6%)	163 (5.1%)	210 (10.4%)	108 (16.2%)	601 (5.8%)	< 0.001
Yes	98 (22.0%)	120 (28.7%)	146 (41.2%)	114 (63.7%)	478 (34.2%)	< 0.001
Shock						
No	140 (3.0%)	147 (4.7%)	147 (8.1%)	69 (13.2%)	503 (5.0%)	< 0.001
Yes	78 (20.9%)	136 (26.4%)	209 (37.7%)	153 (47.4%)	576 (32.6%)	< 0.001
Cardiogenic Shock						
No	160 (3.4%)	177 (5.5%)	179 (9.3%)	98 (17.0%)	614 (5.9%)	< 0.001
Yes	58 (19.7%)	106 (25.6%)	177 (40.0%)	124 (45.9%)	465 (32.7%)	< 0.001
Sepsis						
No	186 (3.8%)	221 (6.6%)	270 (12.7%)	174 (23.4%)	851 (7.7%)	< 0.001
Yes	32 (21.1%)	62 (25.1%)	86 (34.7%)	48 (46.6%)	228 (30.4%)	< 0.001
Respiratory Failure						
No	83 (2.0%)	92 (3.5%)	130 (8.0%)	76 (14.6%)	381 (4.3%)	< 0.001
Yes	135 (16.5%)	191 (19.5%)	226 (30.1%)	146 (44.8%)	698 (24.3%)	< 0.001

ACS						
No	134 (4.7%)	176 (8.3%)	200 (14.9%)	123 (25.5%)	633 (9.3%)	< 0.001
Yes	84 (3.9%)	107 (7.2%)	156 (15.1%)	99 (27.1%)	446 (8.8%)	< 0.001
CHF						
No	82 (2.8%)	95 (5.4%)	124 (12.3%)	76 (22.6%)	377 (6.2%)	< 0.001
Yes	136 (6.7%)	188 (10.1%)	232 (17.0%)	146 (28.6%)	702 (12.2%)	< 0.001

Abbreviations
ACS: acute coronary syndrome
CHF: congestive heart failure

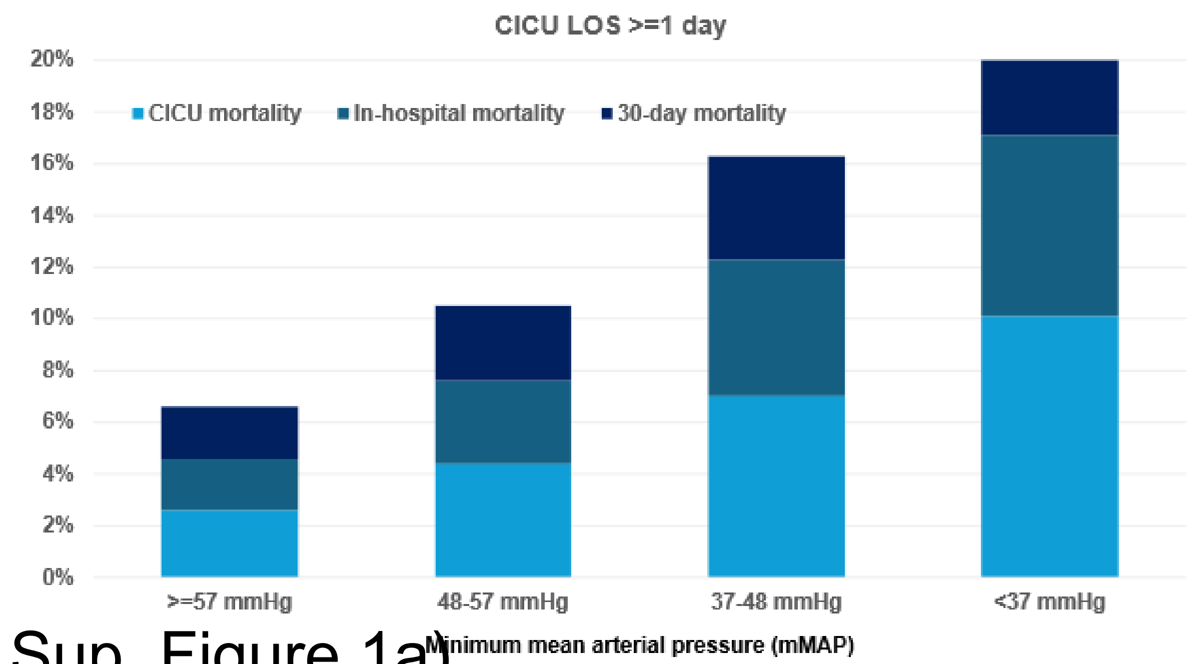

Sup. Figure 1a)

Supplemental Figure 1: Sensitivity analysis demonstrating mortality in patients with ICU length of stay >1day (a), <1day (b), and excluding early CICU deaths (c). *Denotes that 30-day mortality in the CICU length of stay <1 day group with minMAP <37 mmHg was lower than in-hospital mortality.

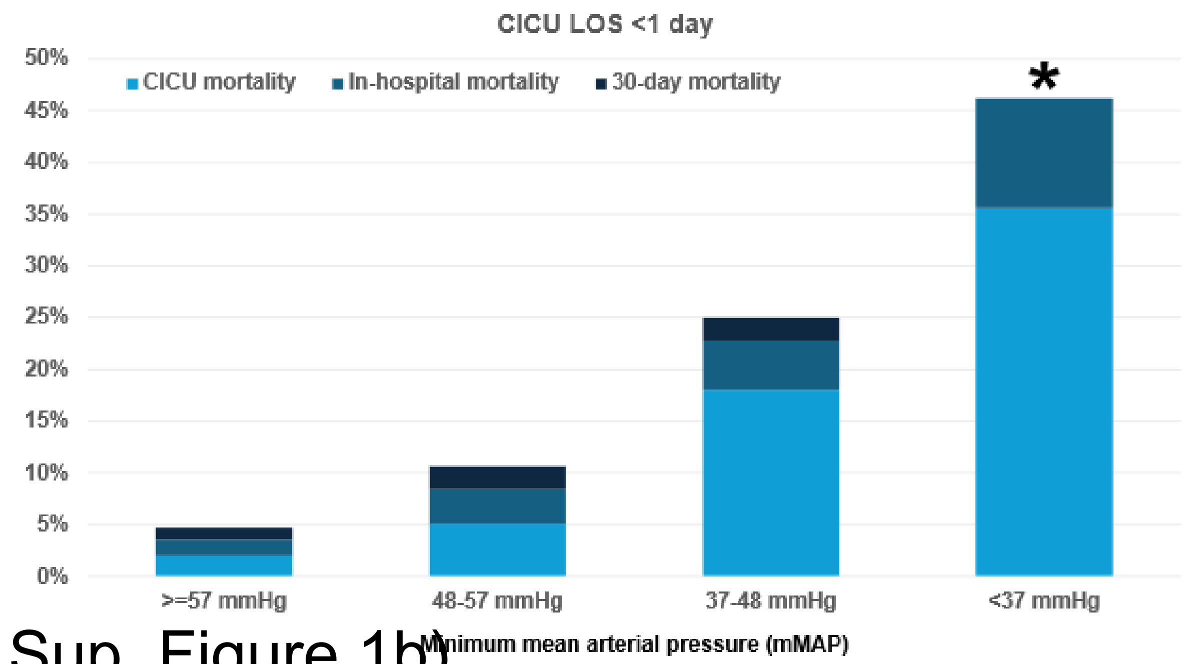

Sup. Figure 1b)

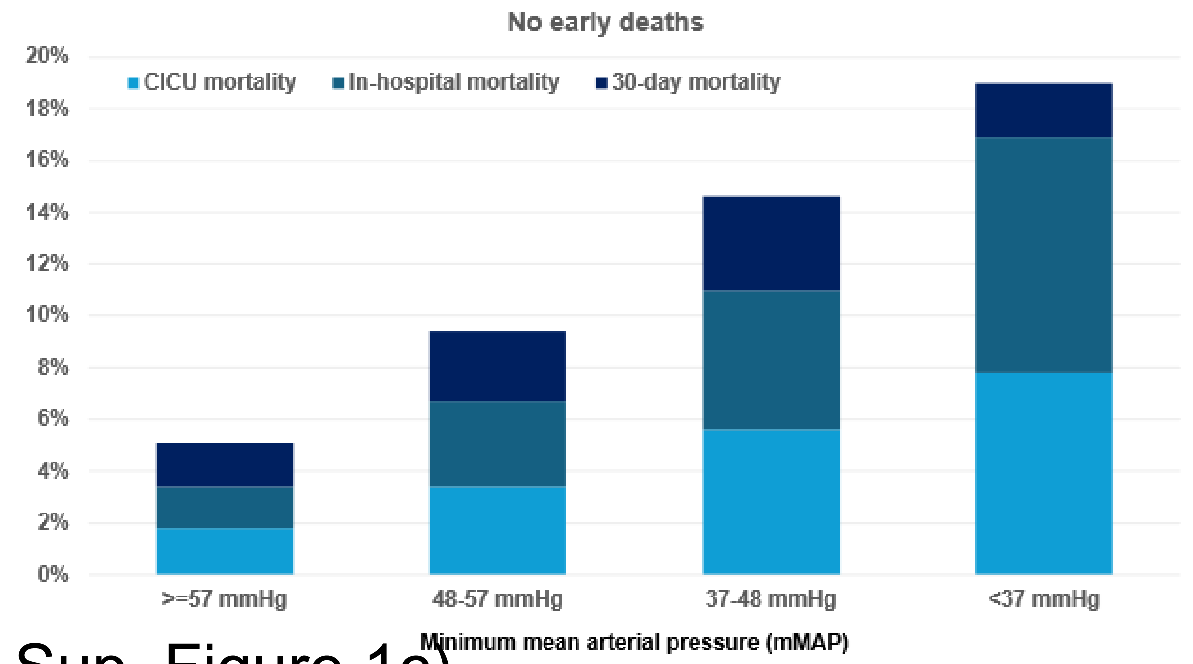

Sup. Figure 1c)

©2025 Mayo Foundation for Medical Education and Research | slide-1

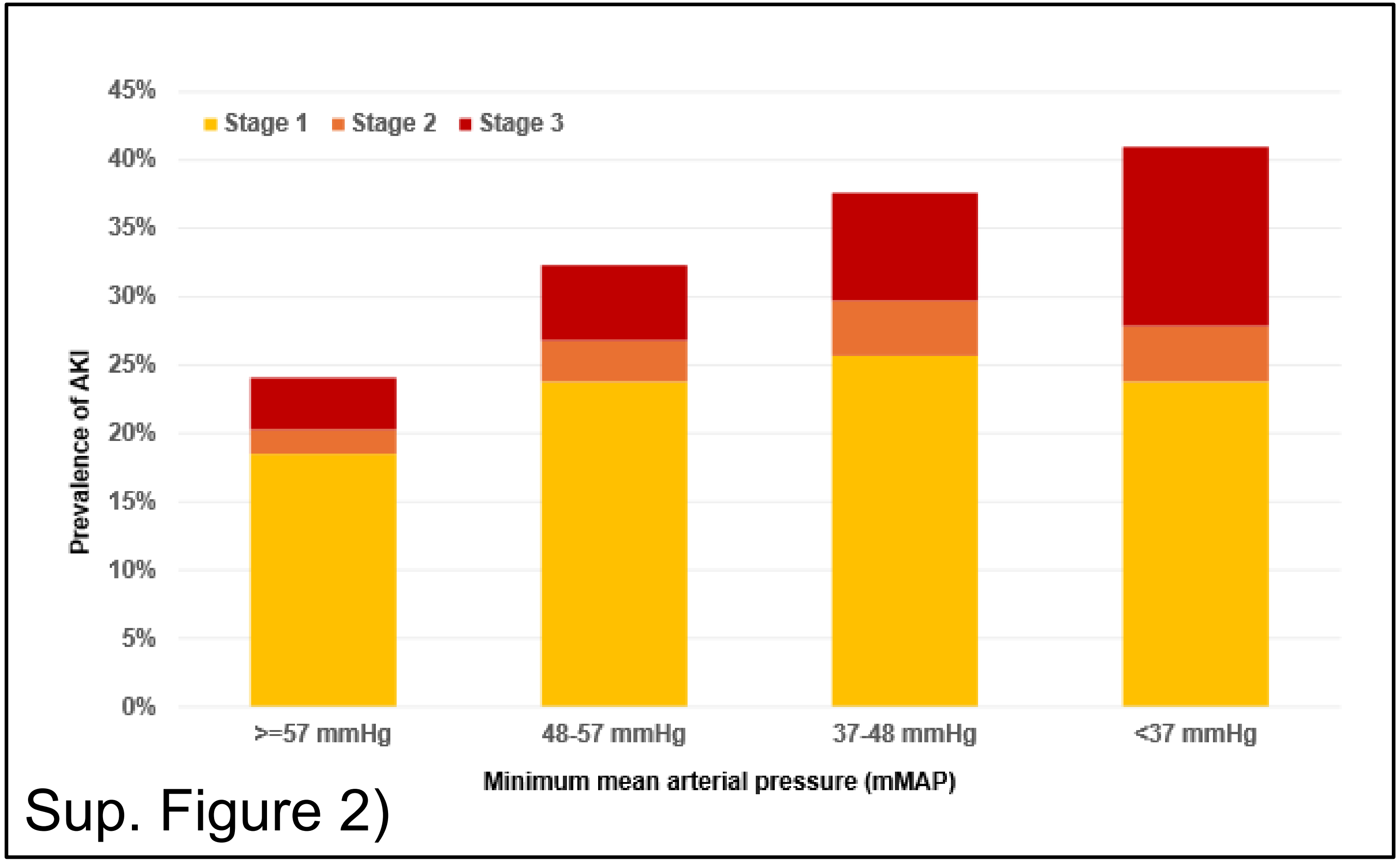

Supplemental Figure 2: Prevalence and severity of AKI by minMAP.

©2025 Mayo Foundation for Medical Education and Research | slide-2
